# Development and validation of a gradient boosting machine to predict prognosis after liver resection for intrahepatic cholangiocarcinoma

**DOI:** 10.1186/s12885-022-09352-3

**Published:** 2022-03-11

**Authors:** Gu-Wei Ji, Chen-Yu Jiao, Zheng-Gang Xu, Xiang-Cheng Li, Ke Wang, Xue-Hao Wang

**Affiliations:** 1 Hepatobiliary Center, The First Affiliated Hospital of Nanjing Medical University, Nanjing, People’s Republic of China; 2 Key Laboratory of Liver Transplantation, Chinese Academy of Medical Sciences, Nanjing, People’s Republic of China; 3 NHC Key Laboratory of Living Donor Liver Transplantation, Nanjing Medical University), Nanjing, People’s Republic of China

**Keywords:** Intrahepatic cholangiocarcinoma, Machine learning, Survival, Modelling, Surgery

## Abstract

**Background:**

Accurate prognosis assessment is essential for surgically resected intrahepatic cholangiocarcinoma (ICC) while published prognostic tools are limited by modest performance. We therefore aimed to establish a novel model to predict survival in resected ICC based on readily-available clinical parameters using machine learning technique.

**Methods:**

A gradient boosting machine (GBM) was trained and validated to predict the likelihood of cancer-specific survival (CSS) on data from a Chinese hospital-based database using nested cross-validation, and then tested on the Surveillance, Epidemiology, and End Results (SEER) database. The performance of GBM model was compared with that of proposed prognostic score and staging system.

**Results:**

A total of 1050 ICC patients (401 from China and 649 from SEER) treated with resection were included. Seven covariates were identified and entered into the GBM model: age, tumor size, tumor number, vascular invasion, number of regional lymph node metastasis, histological grade, and type of surgery. The GBM model predicted CSS with C-Statistics ≥ 0.72 and outperformed proposed prognostic score or system across study cohorts, even in sub-cohort with missing data. Calibration plots of predicted probabilities against observed survival rates indicated excellent concordance. Decision curve analysis demonstrated that the model had high clinical utility. The GBM model was able to stratify 5-year CSS ranging from over 54% in low-risk subset to 0% in high-risk subset.

**Conclusions:**

We trained and validated a GBM model that allows a more accurate estimation of patient survival after resection compared with other prognostic indices. Such a model is readily integrated into a decision-support electronic health record system, and may improve therapeutic strategies for patients with resected ICC.

**Supplementary Information:**

The online version contains supplementary material available at 10.1186/s12885-022-09352-3.

## Background

Intrahepatic cholangiocarcinoma (ICC) ranks as the second most common primary liver cancer after hepatocellular carcinoma. The increasing incidence and accompanying rising mortality rates of ICC over the past few decades worldwide have become a significant healthcare problem [1]. Although surgery offers the best chance of a potential cure for patients with localized and resectable ICC, the prognosis following resection remains discouraging, with 5-year survival of 25–35%, and mortality largely attributes to tumor recurrence, with 50–70% of patients experiencing tumor recurrence [2–4]. Thus, accurate prognosis assessment is essential to help direct appropriate individualized treatment for surgically resected ICC and thereafter optimize outcomes.

The American Joint Committee on Cancer (AJCC) staging manual represents the most widely used system for surgically managed patients with ICC. Although constantly refined, the AJCC staging system exhibits modest prognostic accuracy for resected cases and the prognosis of patients with the same stage varies [2, 5]. By using data from institutional series, multiple prognostic nomograms have been established to predict survival after resection for ICC [2, 6]. Recently, Raoof et al. [7] developed a prognostic score for ICC based on the independent association of multifocality, extrahepatic extension, grade, nodal status, and age (MEGNA) with survival using cases derived from a population-based database. All these published models were developed on factors known after surgery because several determinants, such as tumor grade and nodal status, can be ascertained only in the postoperative context. However, all these models are outmoded and rigid tools by nature because all variables were examined by Cox proportional hazard regression and assigned fixed weights, and missing data are not allowed. Hence, new methods to improve survival estimation and goal-concordant cancer care are warranted.

Today, machine learning (ML) algorithms enable computers to learn from large-scale, heterogeneous health-care data without predefined rules. ML models have offered considerable advantages over traditional statistical models for many tasks, such as diagnosis and classification, risk stratification, and survival prediction [8]. Unfortunately, many popular ML algorithms are essentially black boxes that limit the physician’s trust in their results. Gradient boosting machine (GBM) is currently considered as the state-of-the-art algorithm for prediction with tabular data and has been consistently utilized as the top performer of modelling competitions in a variety of clinical scenarios [9–11]. GBM algorithm can be disassembled into simple decision-tree-base-learners, which provide model-centric explanations, and handle missing values with the gradient-boosting predictor. To date, there has been no effort to use GBM to take full advantage of readily-available clinical information to help physicians predict survival of patients with resected ICC. Accordingly, we assembled a large-scale international cohort of ICC patients to design and evaluate a GBM model for prognosis prediction. We hypothesized that this model would outperform routinely used or previously established prognostic indices in ICC.

## Methods

### Patient population and study design

Adult patients (age ≥ 20 years) with histology-confirmed ICC who underwent liver resection were retrospectively identified from two sources: (1) consecutive patients treated between 2009 and 2019 at the First Affiliated Hospital of Nanjing Medical University (FAHNJMU) (Nanjing, China); (2) patients (histology codes 8140 and 8160 for adenocarcinoma and cholangiocarcinoma in combination with site code C22.1 for intrahepatic bile duct, according to International Classification of Diseases for Oncology, 3rd Edition) [12] between 2004 and 2015 in the Surveillance, Epidemiology, and End Results (SEER) database. The exclusion criteria were: (1) loss to follow-up or a survival of < 1 month; (2) missing information on the type of resection; (3) another malignant primary tumor prior to ICC diagnosis; (4) cause of death unknown; (5) exact tumor size unknown; (6) incomplete information on tumor extension or metastasis for 8th AJCC staging; (7) distant metastatic disease.

**Fig. 1:**
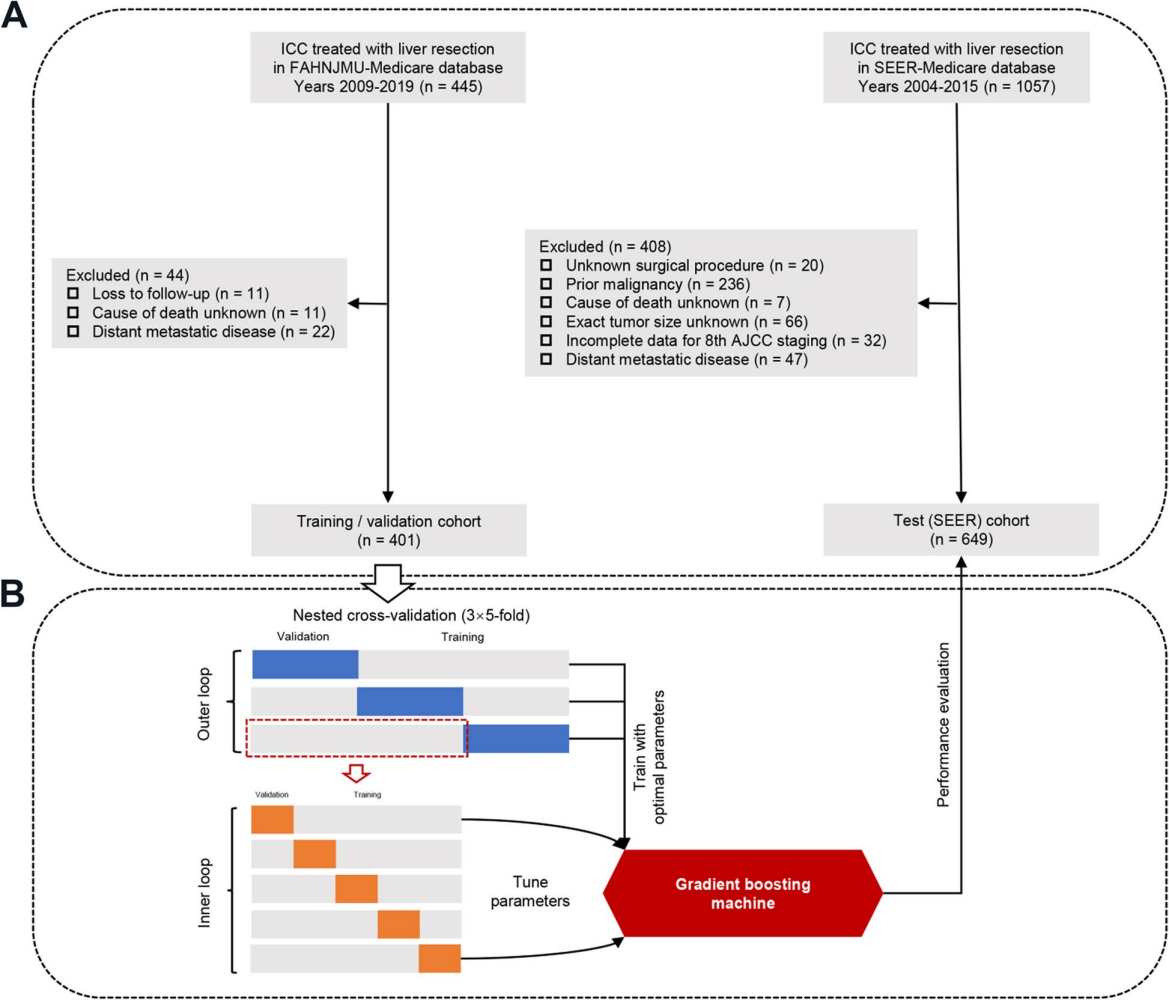
Study flowchart and methodology. **A** Flow chart of the study population. **B** Pipeline to train, validate and test the gradient boosting machine. ICC, Intrahepatic cholangiocarcinoma; FAHNJMU, First Affiliated Hospital of Nanjing Medical University; SEER, Surveillance, Epidemiology, and End Results; AJCC, American Joint Committee on Cancer

The GBM model was trained and validated on data from FAHNJMU using nested cross-validation, and then tested on the SEER database (Fig. 1A). Because the model was developed on the dataset of Asian patients, use of the geographically distinct population from SEER should provide an appropriate assessment for its generalization ability. This study followed the Transparent Reporting of a Multivariable Prediction Model for Individual Prognosis or Diagnosis guideline [13]. This study was approved by the ethics committee of FAHNJMU (Nanjing, China) and the requirement of informed patient consent was waived.

### Data collection and outcome

The pertinent demographic and clinicopathological data were abstracted based on a standardized template. Data collection included the following characteristics of interest: age, gender, tumor size, tumor number, vascular invasion, regional lymph node metastasis (LNM), number of regional LNM, histological grade, visceral peritoneum invasion, adjacent organ invasion, liver fibrosis score, and type of surgery. The above-mentioned covariates are readily retrieved from electronic medical records and routine clinical practice. Patients in the FAHNJMU database were monitored after surgery with laboratory and imaging studies, including liver function, serum tumor markers, ultrasonography, dynamic computed tomography or magnetic resonance imaging, every 3 months during the first 2 years and every 6 months thereafter; the follow-up was terminated on August 20, 2020. Survival data for the SEER database were estimated using statistics from the US Census Bureau [14]. The primary outcome of this study was cancer-specific survival (CSS), defined as the duration from the date of surgery to the date of death from ICC. All deaths from any other cause were counted as non-cancer-specific and censored at the date of the last follow-up.

### Model training, validating and testing

A GBM model that aggregated multiple predictors was trained to predict the likelihood of survival with decision-tree-base-learners using the “gbm” R package. Each base learner may consist of different predictors; predictors with higher importance are utilized in more decision trees as well as earlier in the boosting algorithm. Hyperparameters were tuned with a grid search approach in a 3 × fivefold nested, cross-validated, manner (3 outer iterations and 5 inner iterations) on the training/validation cohort using the “mlr” R package. Nested cross-validation was applied because it more accurately estimates the independent validation error of the given algorithm on unseen datasets by averaging its performance metrics across folds [15]. Study pipeline is schematically depicted in Fig. 1B. The GBM model was then tested on the patients of the test cohort to determine whether it remains accurate when new data are fed into it. We also compared the performance of GBM model to that of AJCC staging system and previously published MEGNA model.

### Statistical analysis

All statistical analyses were performed using R software version 3.4.4 (www.r-project.org). Categorical variables were presented as number (percentage) and compared using χ2 test. Continuous variables were reported as median (interquartile range) and compared using Mann–Whitney *U* test or Kruskal–Wallis rank test, as appropriate. Survival probabilities and 95% confidence intervals (CI) were estimated using the Kaplan–Meier method and compared by the log-rank test. Model performance was measured by Harrell’s C-statistic and 95% CIs were calculated by bootstrapping. Model calibration was performed by plotting the predicted probabilities versus the observed outcomes. Clinical utility was determined by decision curve analysis that quantifies the net benefit associated with the adoption of the model [16]. By using X-tile software [17], the optimal cut-points of GBM predictions were determined to stratify patients at low, intermediate, or high risk for cancer-specific death. A two-sided *P* < 0.05 was considered statistically significant.

## Results

### Patient data

A total of 1050 patients (401 from the FAHNJMU database and 649 from the SEER database; 559 men [53.2%] and 491 women [46.8%]; median [interquartile range] age, 62.0 [53.0–69.0] years) who met the study criteria formed the original dataset. During a median follow-up of 36.2 months (range, 1.0–165.0 months), 591 cancer-specific deaths (56.3%) occurred; the 2-and 5-year CSS rates were 63.1% and 35.6%, respectively. Comparisons of training/validation (*n* = 401) and test (*n* = 649) cohorts are shown in Table 1.

**Table 1:** Comparison of demographic and clinicopathological characteristics between the training/ validation and test cohorts

Characteristics	Training/validation (*n* = 401)	Test (*n* = 649)	*P*-value
Age, years	60.0 (51.0–66.0)	63.0 (55.0–70.0)	< 0.001
Gender			< 0.001
Female	157 (39.2)	333 (51.3)	
Male	244 (60.8)	316 (48.1)	
Tumor size, cm	5.5 (3.7–7.5)	5.5 (3.5–8.0)	0.605
Tumor number			< 0.001
Single	303 (75.6)	402 (61.9)	
Multiple	98 (24.4)	140 (21.6)	
Unknown	0 (0.0)	107 (16.5)	
Vascular invasion			< 0.001
Negative	269 (67.1)	316 (48.7)	
Microvascular	49 (12.2)	158 (24.3)	
Macrovascular	83 (20.7)	55 (8.5)	
Unknown	0 (0.0)	120 (18.5)	
Regional LNM			0.819
Absent	323 (80.5)	519 (80.0)	
Present	78 (19.5)	130 (20.0)	
Number of regional LNM			0.174
0	323 (80.5)	519 (80.0)	
1–2	48 (12.0)	96 (14.8)	
≥ 3	30 (7.5)	34 (5.2)	
Histological grade			< 0.001
Well to moderate	144 (35.9)	372 (57.3)	
Poorly to undifferentiated	257 (64.1)	194 (29.9)	
Unknown	0 (0.0)	83 (12.8)	
Visceral peritoneum invasion			0.268
No	344 (85.8)	572 (88.1)	
Yes	57 (14.2)	77 (11.9)	
Direct invasion of adjacent organ			0.579
No	363 (90.5)	594 (91.5)	
Yes	38 (9.5)	55 (8.5)	
Fibrosis score			< 0.001
None to moderate fibrosis	269 (67.1)	118 (18.2)	
Severe fibrosis or cirrhosis	132 (32.9)	31 (4.8)	
Unknown	0 (0.0)	500 (77.0)	
Type of surgery			< 0.001
Wedge or segmental resection	209 (52.1)	213 (32.8)	
Lobectomy	53 (13.2)	241 (37.1)	
Extended lobectomy	117 (29.2)	96 (14.8)	
Extrahepatic bile duct resection	22 (5.5)	99 (15.3)	
Median CSS time, months^a^	29.6 (25.9–39.2)	39.0 (35.0–44.0)	0.011

Continuous variables reported as median (interquartile range) and categorical variables reported as number (percentage)

*Abbreviations: LNM* lymph node metastasis, *CSS* cancer-specific survival

^†^*P* value calculated by log-rank test

^a^Numbers in parentheses are 95% confidence interval

### GBM prognostic model

**Fig. 2:**
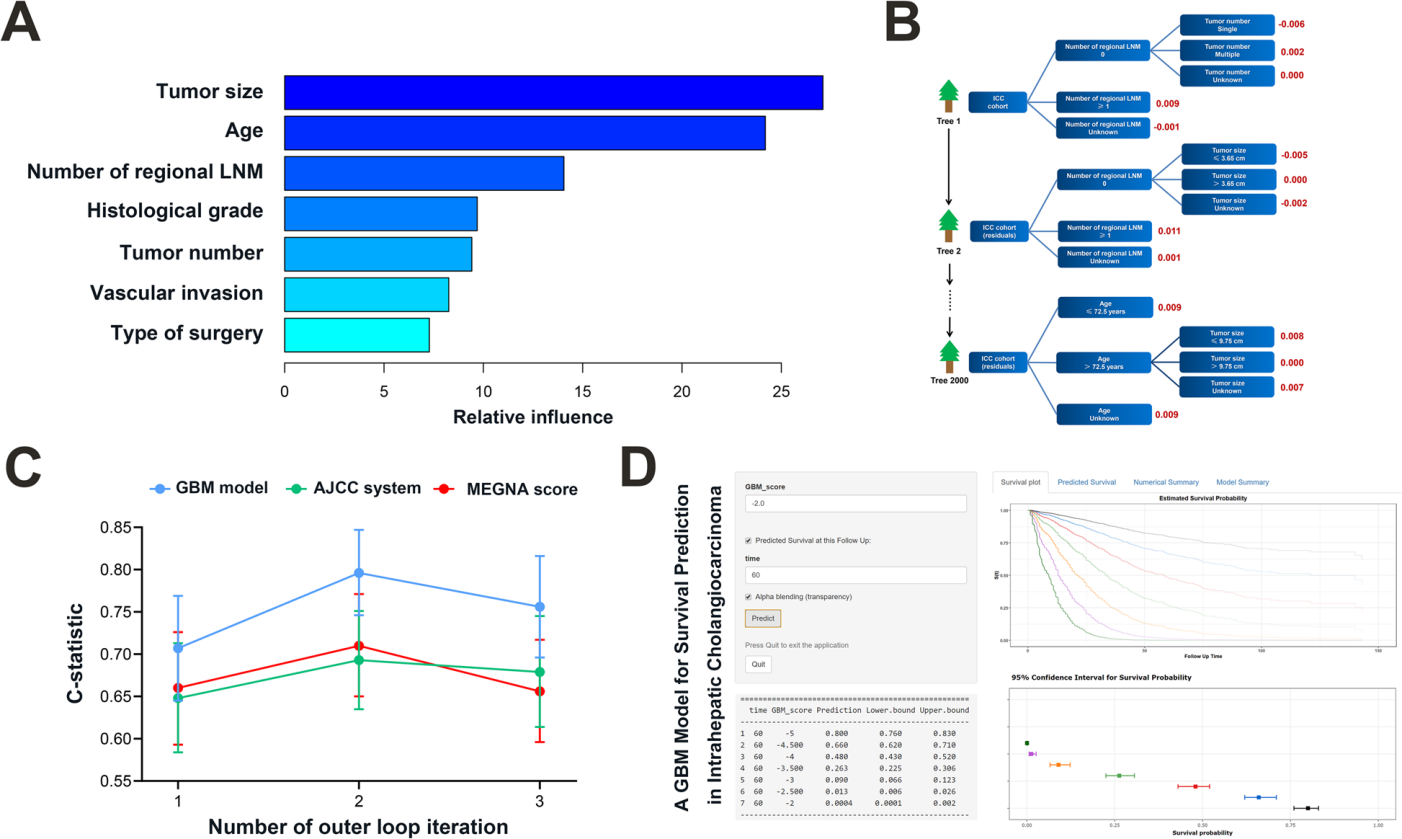
Overview of the gradient boosting machine (GBM) model. **A** Variables included in the model and their relative influence. **B** Illustrative example of the proposed GBM model, which builds the model by combining predictions from stumps of massive decision-tree-base-learners in a step-wise fashion. Prediction score is estimated by adding up the predictions (red number) attached to the terminal nodes of all 2000 decision trees where the patient traverses. **C **Performance of GBM model as compared with that of American Joint Committee on Cancer (AJCC) staging system and multifocality, extrahepatic extension, grade, nodal status, and age (MEGNA) prognostic score in the internal validation group. **D **Online model deployment based on the GBM prediction. LNM, lymph node metastasis

Based on the training/validation cohort, we explored 12 potential model covariates using GBM algorithm and nested cross-validation. We utilized 2000 decision trees sequentially, with at least 5 observations in each terminal node; the decision tree depth was optimized at 2, corresponding to 2-way interactions, and the shrinkage parameter was optimized at 0.01. Covariates with a relative influence greater than 6 (age, tumor size, tumor number, vascular invasion, number of regional LNM, histological grade, and type of surgery) were integrated into the GBM model developed to predict CSS (Fig. 2A-B**).** The most important feature in the GBM model was tumor size, followed by patient age and number of regional LNM. No difference was observed with regard to GBM prediction scores between training/validation and test cohorts (*P* = 0.499) (Fig. S1).

### Model performance

**Fig. 3:**
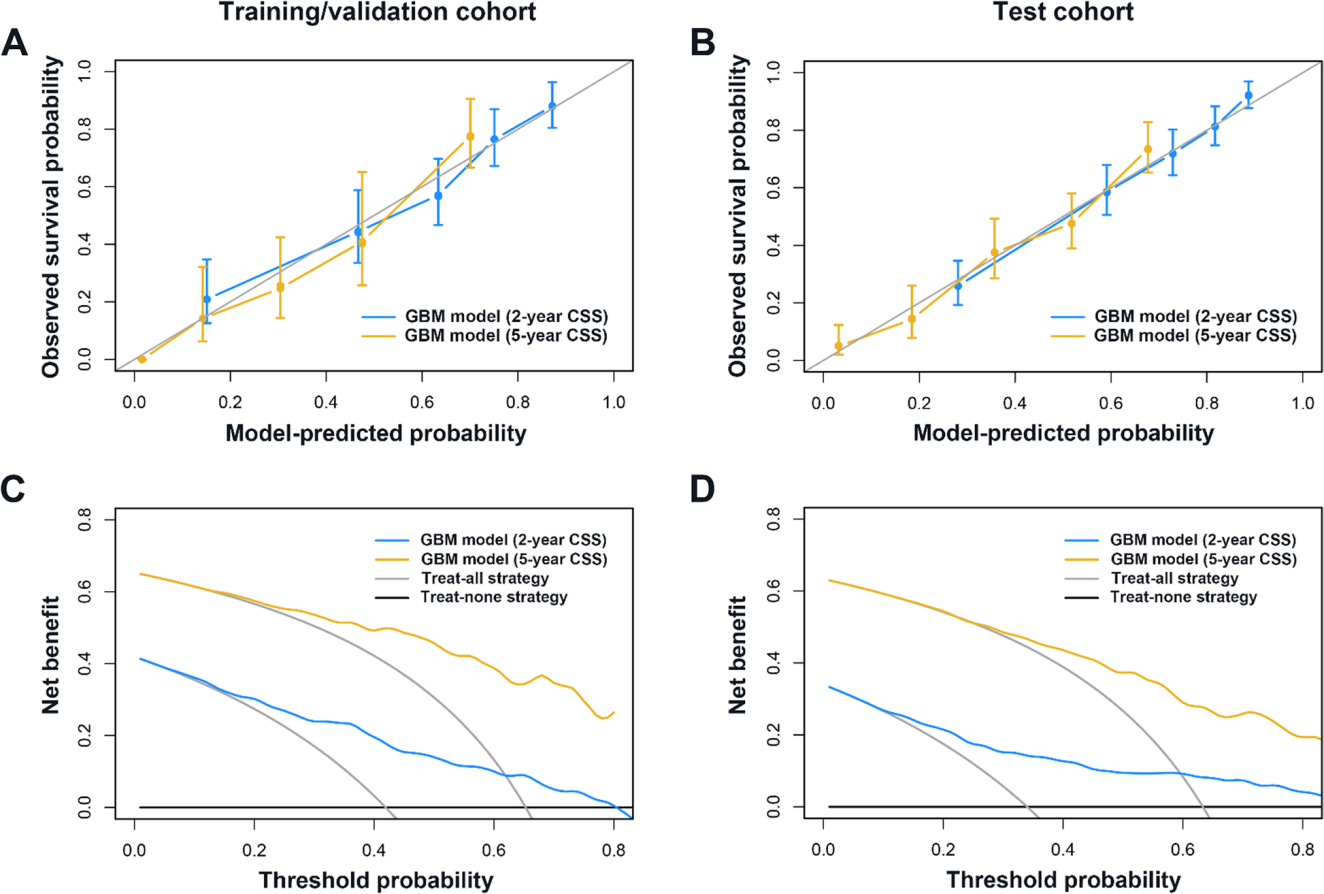
Calibration and clinical utility of the gradient boosting machine (GBM) model. Calibration curves of predicted compared with observed CSS probability at 2 and 5 years in the training/validation **A** and the test **B** cohort. Decision curve analysis comparing the model with other strategies for predicting 2-and 5-year CSS in the training/validation **C** and the test **D** cohort. The y-axis measures the net benefit at a given threshold probability, which is estimated by summing the benefits (true-positive results) and subtracting the harms (false-positive results), weighting the latter by a factor related to the relative harm of an undetected disease compared with the harm of unnecessary treatment. The gray line represents the treat-all strategy (assuming all die of this disease), and the black line represents the treat-none strategy (assuming none die of this disease). GBM-based model provided greater net benefits compared with other strategies across the majority of threshold probabilities. CSS, cancer-specific survival

For predicting post-resection survival specific for ICC, the GBM model had a C-statistic of 0.751 (95% CI 0.717–0.784) in the training/validation cohort, significantly better than that achieved using 8th edition AJCC criteria as well as MEGNA prognostic score (*P* < 0.001) (Table 2). The internal validation group was the nested cross-validation of the GBM model of the training cohort with approximately 134 patients in each outer loop iteration; GBM model yielded a median C-statistic of 0.756 (range 0.707–0.796) for the composite outcome and outperformed AJCC system (median C-statistic 0.679, range 0.648–0.693,* P* < 0.05) as well as MEGNA score (median C-statistic 0.660, range 0.656–0.710, *P* < 0.05) (Fig. 2C). In the test cohort, the GBM model also offered improved prognostic discrimination (C-statistic, 0.723; 95% CI 0.697–0.749) compared with the AJCC staging system and MEGNA prognostic score (*P* < 0.001) (Table 2). The superior performance of GBM model was further confirmed in sub-cohorts stratified by covariate integrity (complete/missing information) (Table S1). Calibration curves for probability of 2-and 5-year CSS showed excellent agreement between model prediction and actual observation in both the training/validation and test cohorts (Fig. 3A-B). Decision curve analysis demonstrated that GBM model provided larger net benefits to decide which ICC patients to refer to specialized oncological care compared with "treat all" or "treat none" strategy (Fig. 3C-D). We deployed an app (https://machinelearningmodel.shinyapps.io/ICC_App/) that allows real-time survival estimates using the prediction score (Fig. 2D).

**Table 2:** Performance of proposed and existing prognostic tools for ICC

Prognostic tools	C-statistic (95% CI)	*P*-value
Training/validation cohort (*n* = 401)		
GBM model	0.751 (0.717–0.784)	ref
AJCC 8th edition	0.673 (0.637–0.708)	< 0.001
MEGNA prognostic score	0.674 (0.638–0.710)	< 0.001
Test cohort (*n* = 649)		
GBM model	0.723 (0.697–0.749)	ref
AJCC 8th edition	0.636 (0.608–0.664)	< 0.001
MEGNA prognostic score^a^	0.617 (0.582–0.651)	< 0.001

*Abbreviations: ICC* intrahepatic cholangiocarcinoma, *CI* confidence intervals, *GBM* gradient boosting machine, *AJCC* American Joint Committee on Cancer, *MEGNA* multifocality, extrahepatic extension, grade, nodal status, and age, *FAHNJMU* First Affiliated Hospital of Nanjing Medical University, *SEER* Surveillance, Epidemiology, and End Results

^a^Available at baseline (467/649) and compared with GBM model in corresponding sub-cohort

### Risk stratification

**Fig. 4:**
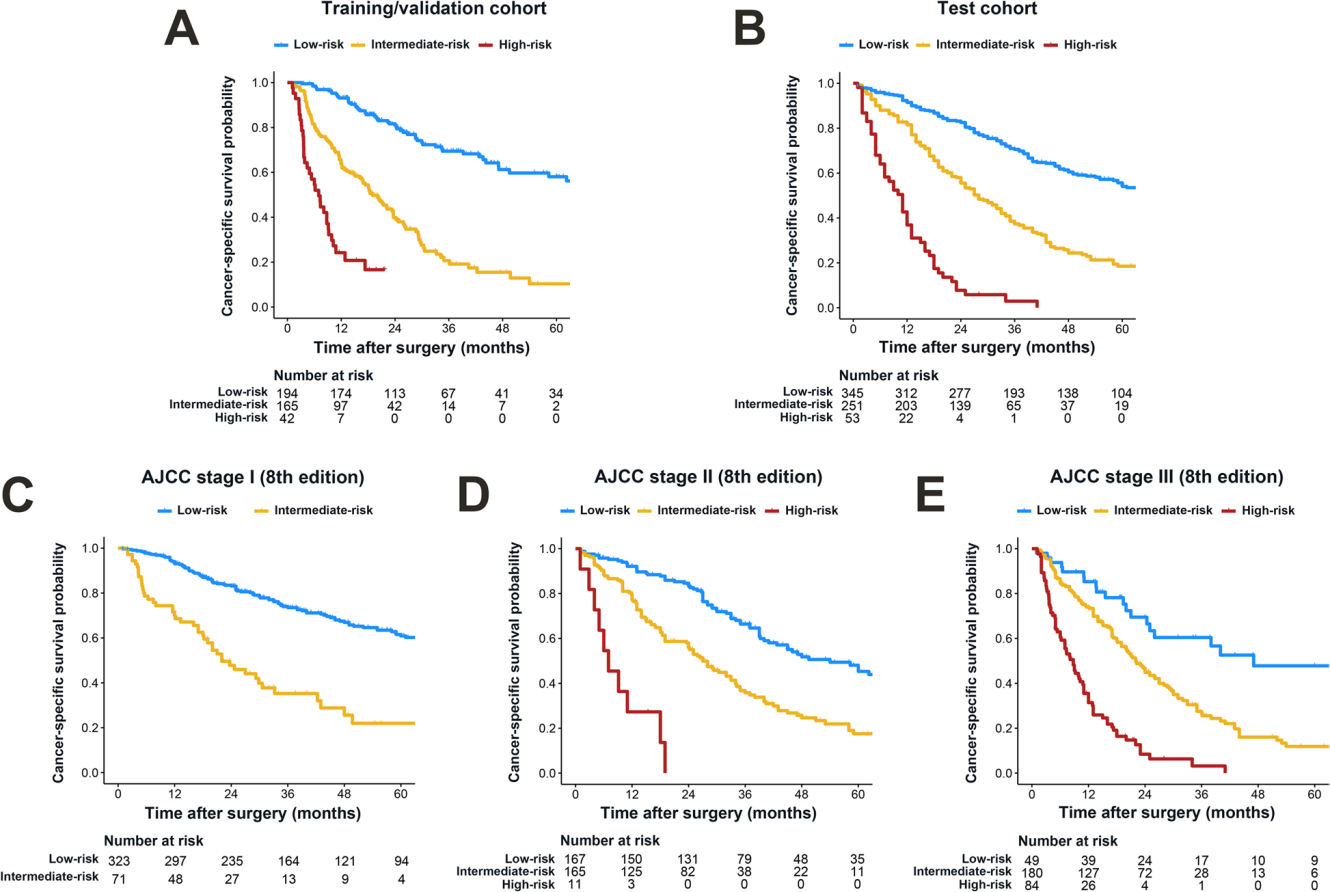
Kaplan–Meier curves demonstrating the differences in cancer-specific survival among low-, intermediate-, and high-risk patients. Survival disparities among different risk groups in the training/validation **A** cohort, the test **B** cohort as well as sub-cohorts stratified by American Joint Committee on Cancer (AJCC) stages **C-E**

With X-tile software identifying optimal cut-off values for prediction scores (-3.65 and -2.45) (Fig. S2), patients were categorized into three groups with a highly different probability of post-resection survival in the training/validation cohort: low risk (194 [48.4%]; 5-year CSS, 58.1%), intermediate risk (165 [41.1%]; 5-year CSS, 10.3%), and high risk (42 [10.5%]; 5-year CSS, not applicable) (*P* < 0.001). The three prognostic strata by using the GBM model were confirmed in the test cohort: low risk (345 [53.1%]; 5-year CSS, 54.1%), intermediate risk (251 [38.7%]; 5-year CSS, 18.5%), and high risk (53 [8.2%]; 5-year CSS, 0.0%) (*P* < 0.001) (Fig. 4A-B; Table 3). Patient characteristics stratified by the GBM model are shown in Table S2. Remarkable differences were observed among three risk groups in all listed characteristics except for patient gender. We also noted that patients were split into distinct prognostic groups across the AJCC stages using the proposed GBM model (*P* < 0.001) (Fig. 4C-E).

**Table 3:** Cancer-specific survival according to risk stratification

Risk group	Median time, months (95% CI)	2-year rate, % (95% CI)	5-year rate, % (95% CI)	Hazard ratio (95% CI)	*P*-value
Training/validation cohort (*n* = 401)					
Low-risk (*n* = 194)	74.6 (58.3-NA)	81.6 (76.0–87.6)	58.1 (49.1–68.6)	1	
Intermediate-risk (*n* = 165)	19.0 (16.6–23.7)	39.9 (32.4–49.1)	10.3 (4.8–22.2)	3.901 (2.826–5.384)	< 0.001^*^
High-risk (*n* = 42)	7.0 (4.4–9.9)	NA	NA	2.794 (1.606–4.863)	< 0.001^†^
Test cohort (*n* = 649)					
Low-risk (*n* = 345)	73.0 (60.0–89.0)	82.5 (78.6–86.7)	54.1 (48.4–60.4)	1	
Intermediate-risk (*n* = 251)	28.0 (24.0–33.0)	55.5 (49.6–62.1)	18.5 (13.3–25.8)	2.496 (1.980–3.146)	< 0.001^*^
High-risk (*n* = 53)	11.0 (7.0–13.0)	7.8 (3.0–19.9)	0.0 (NA)	3.509 (2.149–5.728)	< 0.001^†^

*Abbreviations: CI* confidence intervals, *NA* not applicable

^*^P value versus low-risk; ^†^*P* value versus intermediate-risk

## Discussion

Accurate prediction of survival in ICC is important for decision making and counseling of patients. By harvesting data from over 1000 patients with surgically managed ICC, we trained, validated and tested a novel gradient-boosting ML model that utilized readily available clinical data and provided accurate prognosis prediction (C-statistic ≥ 0.72). The GBM model outperformed both the AJCC staging system as well as the previously published MEGNA score. Importantly, this GBM model increased the number of low-risk/early-stage patients who could be identified by approximately 1.4-fold as compared to the widely adopted AJCC system.

Genomic biomarkers may provide prognostic information; however, their applicability is limited in routine clinical care [18]. Notably, a simple system that utilizes readily available clinical data and provides accurate prognosis estimates remains the preferred reference for personalized management in clinical oncology. Clinicians already use simple models to discuss, for example, the benefit of adjuvant therapy with patients [19]. Prior efforts to develop parsimonious models to predict the prognosis for patients with ICC have mostly been reliant on Cox regression modeling strategies [2, 6, 7]. The Cox model, also known as the proportional hazards model, assumes that the interactions between covariates are homogeneous and different covariates multiplicatively contribute to the hazard function but complex relationships exist between factors related to ICC prognosis [20, 21]. Moreover, Cox regression analysis must be performed in cases with complete information and improper management of data, such as excluding cases with missing data, introduces substantial bias, as noted across various cancer types [22, 23]. In that setting, ML techniques have a significant role to play.

Recent recommendations have emphasized the explainability along with the robustness to incomplete data as the priority in ML research [24, 25]. Decision tree-based algorithms represent a large family of ML techniques. Current machine-based classification and regression trees (CART) have been applied to define prognostic groups for patients with resected ICC because of their simplicity and intuitive interpretation [20, 21]. Nevertheless, such trees suffer from intrinsic limitations in predictive performance. Gradient boosting of regression trees enables highly competitive, robust, interpretable procedures to relax the assumption of proportional hazards and allow for complicated relationships between covariates that improve the predictive accuracy [26]. GBM model can be disassembled into massive decision-tree-base-learners (CART models) so that it is possible to decipher the intrinsical structure of our proposed model and understand how the machine makes predictions. Moreover, GBM algorithm has a built-in functionality to handle missing values that permits utilizing data from, and assigning classification to, all observations in the cohort without the need of imputation for missing data [9]. This considerably broadens the datasets available and the scope for building prognostic models. Another limit to ML techniques is overfitting (low bias and high variance), defined as a superior performance in the training/validation cohort but inferior performance in an independent test dataset [27]. To avoid this issue, a nested cross-validation approach was applied for hyperparameter tuning in this study because it prevents information leaking between cases used for model training and validation [15]. Comparable performance in the training/validation cohort, the test cohort as well as sub-cohorts stratified by covariate integrity further confirmed good reproducibility and reliability of our GBM model.

Although ML algorithms may improve prediction performance in the prognostic setting, it is important to demonstrate that improved accuracy can translate to better clinician and patient decision-making. We therefore provided an app (https://machinelearningmodel.shinyapps.io/ICC_App/) that allows for a GBM prediction input and an immediate feedback of survival probabilities at individualized time scale. Also, the GBM model was able to identify three risk strata for cancer-specific death (ie, low-, intermediate-, and high-risk groups). Nearly half of ICC patients who suffered from extremely dismal prognosis following resection were identified by using the GBM model. Therefore, adjuvant treatments, such as capecitabine-based chemotherapy or immune-directed therapy, is desirable for intermediate-to-high risk patients. In turn, over half of patients with surgically resected ICC were categorized as low risk with satisfactory long-term survival and thus may receive no adjuvant therapy. On the other hand, the GBM model highlights that the number of regional LNM holds more prognostic information compared with the involvement of regional LNM, which is consistent with previous publication [28].

Several limitations warrant attention when interpreting the results of this model. First, our model was developed, validated and tested using retrospective data; a prospective validation study should be conducted to confirm our results prior to its routine use in clinical practice. Nonetheless, the top-ranked features in the proposed model, such as tumor size, number of regional LNM, vascular invasion and tumor number, are all well-established prognostic factors, lending validity to our GBM model [1]. Second, patient and tumor characteristics included in this study were limited because some potential prognostic factors, such as carcinoembryonic antigen, carbohydrate antigen 19–9, surgical margin status and treatment of recurrent disease, were not available in SEER database. However, the seven covariates integrated into our model are readily accessible from health-care data, indicating its simplicity and feasibility; the proposed GBM model is still able to provide accurate prediction and risk stratification even without additional prognostic information. Finally, our GBM model promises to identify ICC patients at high risk for cancer-specific death after resection but does not provide individualized solution for how to manage these patients clinically to ultimately improve prognosis.

## Conclusions

In conclusion, we developed and validated an interpretable ML model using readily available clinical data to predict the prognosis for patients with resected ICC. Our GBM model provides more-accurate determination of survival probabilities compared with previously proposed MEGNA score and widely adopted AJCC staging system. Such an easy-to-use tool may lead to better personalized treatments for patients with resected ICC in future clinical practice.

## Supplementary Information

**Additional file 1:** **Fig.** **S1**. Scatter plot of gradient boosting machine-based prediction scores in the training/validation and test cohort. Scores are reported as median (interquartile range). **Fig.** **S2**. X-tile analysis to determine the optimal cut-points for GBM-based prediction scores. The optimal cut-points highlighted by black circle (A) are detailed in histogram of the training/validation cohort (B) with corresponding Kaplan-Meier curves (C). GBM gradient boosting machine. **Table S1**. Comparison of proposed and existing prognostic tools for ICC in sub-cohort with or without missing covariates. **Table S2**. Comparison of demographic and clinicopathological characteristics among different risk groups

## Data Availability

The data that support the findings of this study are available from the corresponding author upon reasonable request.

## Abbreviations

ICC: Intrahepatic cholangiocarcinoma
AJCC: American Joint Committee on Cancer
MEGNA: Multifocality, extrahepatic extension, grade, nodal status, and age
ML: Machine learning
GBM: Gradient boosting machine
FAHNJMU: First Affiliated Hospital of Nanjing Medical University
SEER: Surveillance, Epidemiology, and End Results
LNM: lymph node metastasis
CSS: Cancer-specific survival
CART: Classification and regression trees
