# Supplementary material for: Development and validation of a gradient boosting machine to predict prognosis after liver resection for intrahepatic cholangiocarcinoma
Source: BMC Cancer. 2022 Mar 11;22:258. doi: 10.1186/s12885-022-09352-3 (PMC8915487; doi:10.1186/s12885-022-09352-3)
Supplement: Supplementary file 1 — Additional file 1: Fig. S1. Scatter plot of gradient boosting machine-based prediction scores in the training/validation and test cohort. Scores are reported as median (interquartile range). Fig. S2. X-tile analysis to determine the optimal cut-points for GBM-based prediction scores. The optimal cut-points highlighted by black circle (A) are detailed in histogram of the training/validation cohort (B) with corresponding Kaplan-Meier curves (C). GBM gradient boosting machine. Table S1. Comparison of proposed and existing prognostic tools for ICC in sub-cohort with or without missing covariates. Table S2. Comparison of demographic and clinicopathological characteristics among different risk groups [file 12885_2022_9352_MOESM1_ESM.docx]

**Additional file 1**

**
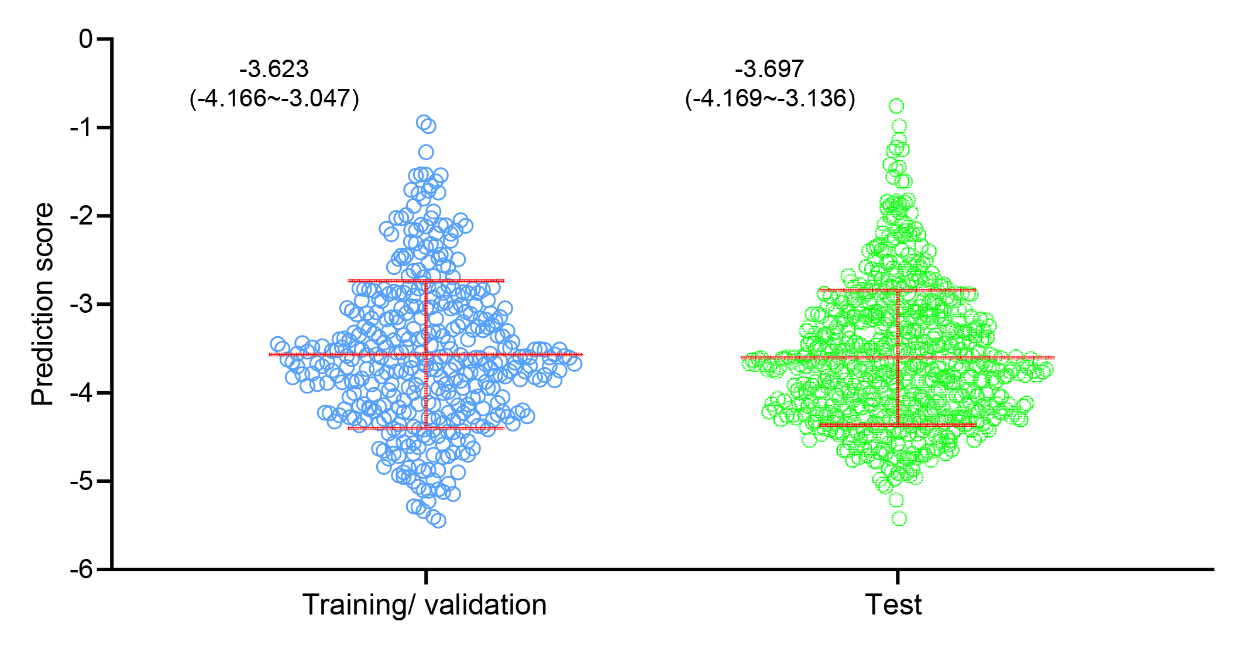
**

**Fig. S1. Scatter plot of gradient boosting machine-based prediction scores in the training/validation and test cohort.** Scores are reported as median (interquartile range).


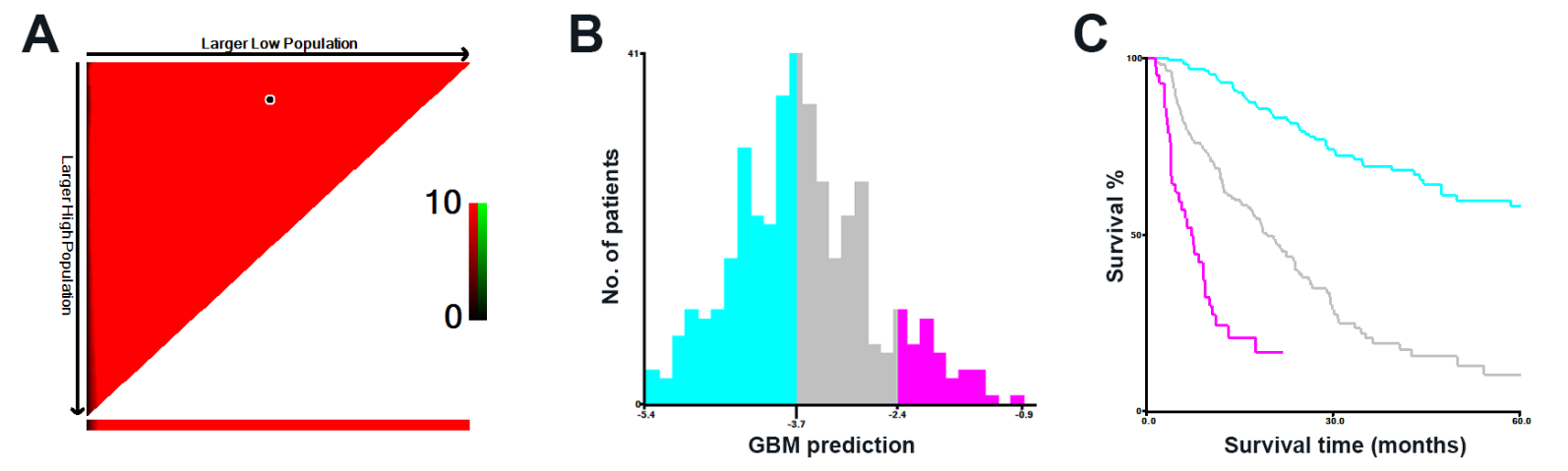


**Fig. S2.** **X-tile analysis to determine the optimal cut-points for GBM-based prediction scores.** The optimal cut-points highlighted by black circle (A) are detailed in histogram of the training/validation cohort (B) with corresponding Kaplan-Meier curves (C). GBM, gradient boosting machine.

**Table S1. Comparison of proposed and existing prognostic tools** **for ICC in sub-cohort with or without missing covariates**

| **Prognostic tools** | **C-statistic (95% CI)** | ***P*-value** |
| --- | --- | --- |
| Sub-cohort with complete covariates (n = 828) ^*^ |  |  |
| GBM model | 0.735 (0.711-0.759) | ref |
| AJCC 8th edition | 0.652 (0.626-0.679) | <0.001 |
| MEGNA prognostic score | 0.652 (0.626-0.677) | <0.001 |
| Sub-cohort with missing covariates (n = 222) ^*^ |  |  |
| GBM model | 0.734 (0.693-0.776) | ref |
| AJCC 8th edition | 0.601 (0.556-0.645) | <0.001 |
| MEGNA prognostic score ^a^ | 0.557 (0.449-0.666) | <0.001 |

^a^ Available at baseline (40/222) and compared with GBM model in corresponding sub-cohort

^*^ Covariates refer to the variables included in the GBM model.

Abbreviations: ICC, intrahepatic cholangiocarcinoma; CI, confidence intervals; GBM, gradient boosting machine; AJCC, American Joint Committee on Cancer; MEGNA, multifocality, extrahepatic extension, grade, nodal status, and age.

**Table S2. Comparison of demographic and clinicopathological characteristics among different risk groups**

| **Characteristics** | **Low-risk**  **(n = 539)** | **Intermediate-risk**  **(n = 416)** | **High-risk**  **(n = 95)** | ***P*-value** |
| --- | --- | --- | --- | --- |
| Age, years | 61.0 (52.0-68.0) | 63.0 (54.0-70.0) | 64.0 (53.0-72.0) | 0.002 |
| Gender |  |  |  | 0.586 |
| Female | 258 (47.9) | 186 (44.7) | 46 (48.4) |  |
| Male | 281 (52.1) | 230 (55.3) | 49 (51.6) |  |
| Tumor size, cm | 4.2 (3.0-6.0) | 6.5 (4.8-9.0) | 7.0 (5.4-10.0) | <0.001 |
| Tumor number |  |  |  | <0.001 |
| Single | 455 (84.5) | 217 (52.2) | 33 (34.7) |  |
| Multiple | 59 (10.9) | 136 (32.7) | 43 (45.3) |  |
| Unknown | 25 (4.6) | 63 (15.1) | 19 (20.0) |  |
| Vascular invasion |  |  |  | <0.001 |
| Negative | 379 (70.3) | 183 (44.0) | 23 (24.2) |  |
| Microvascular | 95 (17.6) | 89 (21.4) | 23 (24.2) |  |
| Macrovascular | 45 (8.4) | 62 (14.9) | 31 (32.6) |  |
| Unknown | 20 (3.7) | 82 (19.7) | 18 (19.0) |  |
| Regional LNM |  |  |  | <0.001 |
| Absent | 531 (98.5) | 298 (71.6) | 13 (13.7) |  |
| Present | 8 (1.5) | 118 (28.4) | 82 (86.3) |  |
| Number of regional LNM |  |  |  | <0.001 |
| 0 | 531 (98.5) | 298 (71.6) | 13 (13.7) |  |
| 1-2 | 8 (1.5) | 97 (23.3) | 39 (41.0) |  |
| ≥3 | 0 (0.0) | 21 (5.1) | 43 (45.3) |  |
| Histological grade |  |  |  | <0.001 |
| Well to moderate | 368 (68.3) | 137 (32.9) | 11 (11.6) |  |
| Poorly to undifferentiated | 132 (24.5) | 245 (58.9) | 74 (77.9) |  |
| Unknown | 39 (7.2) | 34 (8.2) | 10 (10.5) |  |
| Perforation of visceral peritoneum |  |  |  | <0.001 |
| No | 507 (94.1) | 340 (81.7) | 69 (72.6) |  |
| Yes | 32 (5.9) | 76 (18.3) | 26 (27.4) |  |
| Invasion of adjacent organs |  |  |  | <0.001 |
| No | 517 (95.9) | 370 (88.9) | 70 (73.7) |  |
| Yes | 22 (4.1) | 46 (11.1) | 25 (26.3) |  |
| Fibrosis score |  |  |  | 0.025 |
| None to moderate fibrosis | 188 (34.9) | 156 (37.5) | 43 (45.2) |  |
| Severe fibrosis or cirrhosis | 100 (18.5) | 56 (13.5) | 7 (7.4) |  |
| Unknown | 251 (46.6) | 204 (49.0) | 45 (47.4) |  |
| Type of surgery |  |  |  | <0.001 |
| Wedge or segmental resection | 245 (45.5) | 148 (35.6) | 29 (30.5) |  |
| Lobectomy | 157 (29.1) | 116 (27.9) | 21 (22.1) |  |
| Extended lobectomy | 91 (16.9) | 101 (24.3) | 21 (22.1) |  |
| Extrahepatic bile duct resection | 46 (8.5) | 51 (12.2) | 24 (25.3) |  |

Continuous variables reported as median (interquartile range) and categorical variables reported as number (percentage).

Abbreviations: LNM, lymph node metastasis.
